# Structural insights into the catalytic and inhibitory mechanisms of the flavin transferase FmnB in *Listeria monocytogenes*

**DOI:** 10.1002/mco2.99

**Published:** 2022-01-10

**Authors:** Yanhui Zheng, Weizhu Yan, Chao Dou, Dan Zhou, Yunying Chen, Ying Jin, Lulu Yang, Xiaotao Zeng, Wei Cheng

**Affiliations:** ^1^ Division of Respiratory and Critical Care Medicine Respiratory Infection and Intervention Laboratory of Frontiers Science Center for Disease‐Related Molecular Network State Key Laboratory of Biotherapy West China Hospital of Sichuan University Chengdu China

**Keywords:** catalytic mechanism, divalent metal ion, extracellular electron transfer, FMN transferase FmnB, inhibitory mechanism, listeria monocytogenes

## Abstract

*Listeria monocytogenes*, a food‐borne Gram‐positive pathogen, often causes diseases such as gastroenteritis, bacterial sepsis, and meningitis. Newly discovered extracellular electron transfer (EET) from *L. monocytogenes* plays critical roles in the generation of redox molecules as electron carriers in bacteria. A Mg^2+^‐dependent protein flavin mononucleotide (FMN) transferase (FmnB; UniProt: LMRG_02181) in EET is responsible for the transfer of electrons from intracellular to extracellular by hydrolyzing cofactor flavin adenine dinucleotide (FAD) and transferring FMN. FmnB homologs have been investigated in Gram‐negative bacteria but have been less well studied in Gram‐positive bacteria. In particular, the catalytic and inhibitory mechanisms of FmnB homologs remain elusive. Here, we report a series of crystal structures of apo‐FmnB and FmnB complexed with substrate FAD, three inhibitors AMP, ADP, and ATP, revealing the unusual catalytic triad center (Asp301‐Ser257‐His273) of FmnB. The three inhibitors indeed inhibited the activity of FmnB in varying degrees by occupying the binding site of the FAD substrate. The key residue Arg262 of FmnB was profoundly affected by ADP but not AMP or ATP. Overall, our studies not only provide insights into the promiscuous ligand recognition behavior of FmnB but also shed light on its catalytic and inhibitory mechanisms.

## Graphical abstract

Flavin transferase FmnB is a key protein of the extracellular electron transfer pathway in Listeria monocytogenes, which can hydrolyze flavin adenine dinucleotide and transfer flavin mononucleotide. We analyzed the apo form of the enzyme and the complex structure of the binding substrate, as well as the complex structures of the binding three inhibitors, and identified key residues that affect enzyme activity.

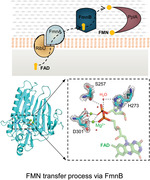

## 1 INTRODUCTION

*Listeria monocytogenes*, as a food‐borne pathogen, often causes severe gastroenteritis, bacterial sepsis, meningitis, and high mortality among infected individuals.^1^, ^2^ At present, there have been considerable research on the metabolism, pathogenicity, interspecific communication, drug resistance, and other aspects of this bacterium. Recently, a novel flavin‐based extracellular electron transfer (EET) pathway was reported.^3^ EET, which is associated with electron transfer from the cytosol to the extracellular, is ubiquitous in electroactive microorganisms (EAMs) and closely related to the metabolism of microorganisms, and it is commonly used in antibiotic drug development.^4^, ^5^, ^6^, ^7^, ^8^

**FIGURE 1:**
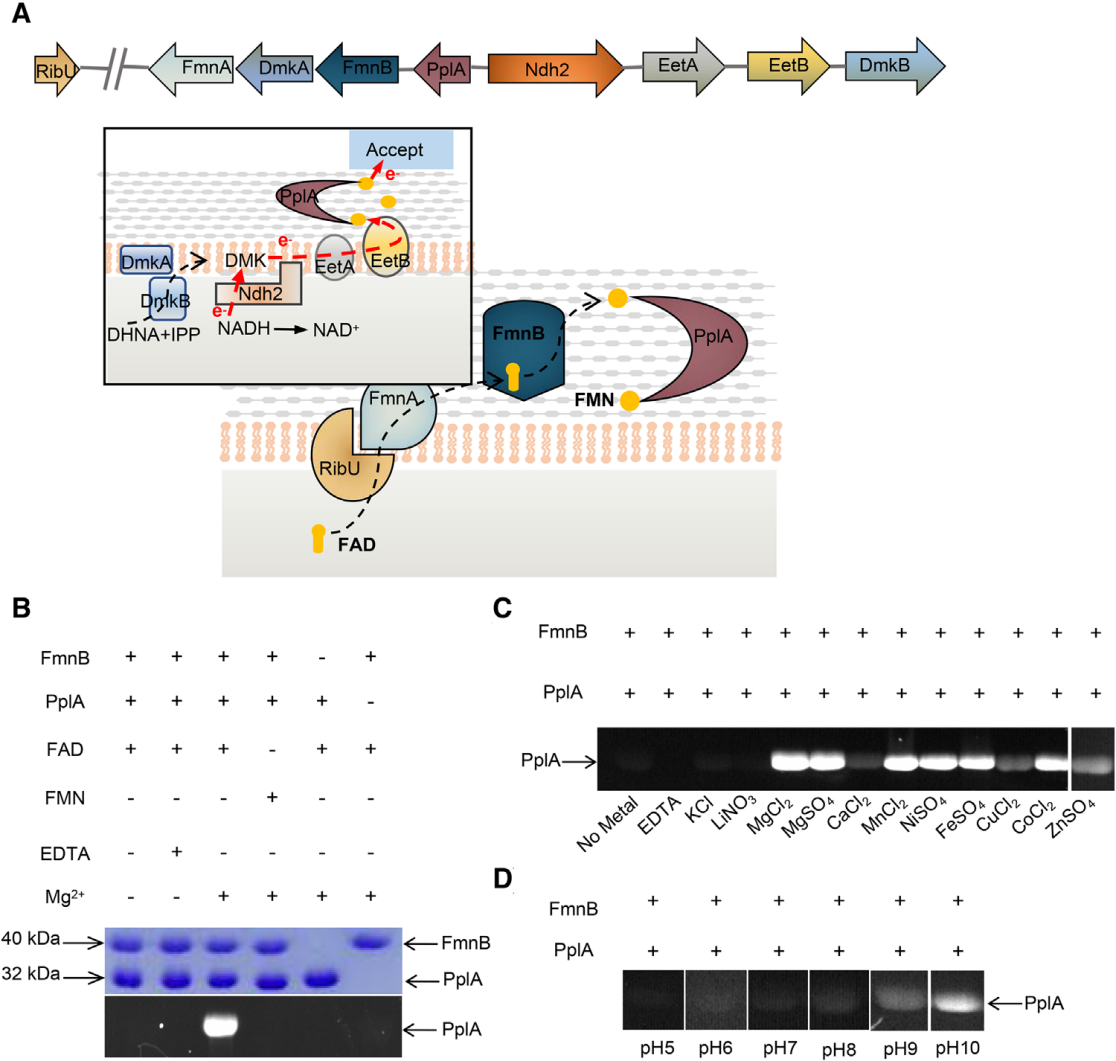
FMN transferase activity of FmnB. A, Model of the molecular basis of EET. EET is achieved by a series of electron transfers. FmnB hydrolyzes FAD and posttranslationally modifies PplA to facilitate electron transfer out of the cell. DMK, demethylmenaquinone; DHNA, 1,4‐dihydroxy‐2‐naphthoyl‐CoA; IPP, isopentenyl pyrophosphate. B, Analysis of FmnB substrate specificity. SDS‐PAGE of recombinant PplA after incubation under specified conditions. Ultraviolet illumination of the gel (bottom) enables visualization of the protein with covalently bound flavin. C, Activation and metal dependence of FmnB activity in the presence of added FAD. The concentration of metal ions used in the experiment was 5 mM. D, pH dependence of FmnB activity in the presence of added Mg^2+^ (5 mM) and FAD

The different characteristics of the cell membrane and cell wall among microorganisms has resulted in the diversity of EET pathways.^4^, ^5^, ^6^ In Gram‐negative bacteria, multiple pathways have been discovered, such as metal reduction (Mtr) pathway composed of a variety of Mtr protein, and the pathway containing MtoAB‐CymA gene cluster.^4^, ^5^, ^6^ Compared with the Gram‐negative bacteria, which have a cell envelope, Gram‐positive bacteria have a thick, nonconductive peptidoglycan layer (30–100 nm), which hinders the EET process and its identification in those bacteria.^9^, ^10^, ^11^ In *L. monocytogenes*, the flavin‐based EET pathway consists of eight proteins (Figure 1A), namely, FmnA, DmkA, FmnB, PplA, Ndh2, EetA, EetB, and DmkB.^3^ These genes are common in many pathogenic bacteria and intestinal microorganisms. Bioinformatics analysis showed that the divergence of the EET pathways between Gram‐positive and Gram‐negative bacteria occurs in the process of electron transfer. To clarify this divergence, we investigated the detailed mechanism of FmnB (protein FMN transferase; UniProt: LMRG_02181) in *L. monocytogenes*, as this protein plays a critical role in FAD hydrolysis during electron shuttling.

FmnB is a flavin transferase belonging to ApbE family; it hydrolyzes flavin adenine dinucleotide (FAD) and converts flavin mononucleotide (FMN) to pheromone lipoprotein (PplA; UniProt: LMRG_02182), and divalent cations are required this process.^3^ To date, some relevant homologous proteins have been reported in *Treponema pallidum* (TpFmnB),^12^ *Pseudomonas stutzeri* (PsFmnB),^13^ and *Vibrio cholerae* (VcFmnB).^14^ However, these homologous proteins are concentrated in Gram‐negative bacteria and have not been reported in Gram‐positive bacteria. FmnB shares only 30% sequence identity with members of ApbE family (Figure S1), although their structures adopt a similar tunnel fold. TpFmnB and VcFmnB have been reported to form a complex structure consisting of an enzyme complexed with the substrate FAD. However, the details of the enzymatic catalysis mechanisms in this family are still unclear, and whether the structure and mechanism of flavin transferase in Gram‐positive and Gram‐negative bacteria are the same remains to be studied. Notably, it was reported^12^, ^13^, ^15^ that the hydrolytic activity of TpFmnB could be inhibited by adenosine triphosphate (ATP), adenosine diphosphate (ADP), and adenosine monophosphate (AMP), and ATP showed the strongest inhibitory effect. However, ADP could promote the catalytic activity for another homolog VcFmnB. Intriguingly, the inhibition or catalytic mechanism remains unclear.

In the current work, we report several crystal structures of FmnB in its apo form and in complexes with its substrate FAD and inhibitors AMP, ADP, and ATP. Based on the structural and biochemical analysis, the key residues were identified, which were involved in the binding of the substrate and the catalytic reaction. Unprecedentedly, we reveal a typical catalytic triad (Asp301‐Ser257‐His273) in FmnB that is involved in flavin transfer and electron transfer, and we propose a possible mechanism for FmnB‐ catalyzed flavin transfer. Subsequently, the enzymatic assays indicated that AMP, ADP and ATP could inhibit FmnB activity. Curiously, ADP exhibited a stronger inhibition ability than AMP and ATP. Notably, the structures of FmnB in complex with distinct inhibitors, together with biochemical assays, revealed that all three inhibitors indeed inhibited the activity of FmnB by occupying the binding site of the FAD substrate. Remarkably, the structures revealed that the key residue Arg262 of FmnB was profoundly affected by ADP but not AMP or ATP. Collectively, our study provides insights into the catalytic and inhibitory mechanisms of FmnB, offering theoretical applications for the development of specific inhibitors against Gram‐positive pathogens.

## 2 RESULTS

### 2.1 FMN transferase activity of FmnB

Metal ions are important in the regulation of most biological responses. These ions are involved in redox and nonredox catalysis.^16^ They act as cofactors to modulate catalytic activity by exploiting different physical characteristics such as ion size and coordination number. To explore the mechanism of action of FmnB, we initially executed an enzymatic assay to characterize the FMNylation activity of FmnB. The results demonstrated that FmnB could catalyze the FMNylation of PplA and confirmed that the enzyme specifically uses FAD as a substrate (Figure 1B), which is consistent with a previous study.^3^ Next, to determine whether the FMNylation activity of FmnB could be impacted by divalent metal ions, we executed an enzymatic assay by adding diverse divalent ions (Figure 1C). As expected, FmnB could indeed catalyze the hydrolysis reaction with several divalent metal cations, such as Mg^2+^, Mn^2+^, Ni^2+^, Fe^2+^, and Co^2+^. However, some divalent metal ions, such as Ca^2+^, Cu^2+^, and Zn^2+^, are less effective in activating FmnB activity than these ions. Notably, K^+^, Li^+^, and Na^+^ do not seem to act as cofactors to activate the catalytic activity of the enzyme. The results indicate that specific divalent cations are essential as cofactors required for efficient catalysis by FmnB.

As previously mentioned, pH was shown to affect the enzymatic activity of FmnB homologs in previous studies.^12^, ^13^, ^15^ To determine the optimal pH, a range of pH values were tested, and the results showed optimal FmnB activity under alkaline conditions (pH 10) (Figure 1D). This is probably because an alkaline environment has to be maintained for the dissociation of arginine and lysine residues at the active site of this enzyme. To explore the mechanism, structural studies were conducted.

### 2.2 Structures of the FmnB protein alone and in complex with the substrate

**FIGURE 2:**
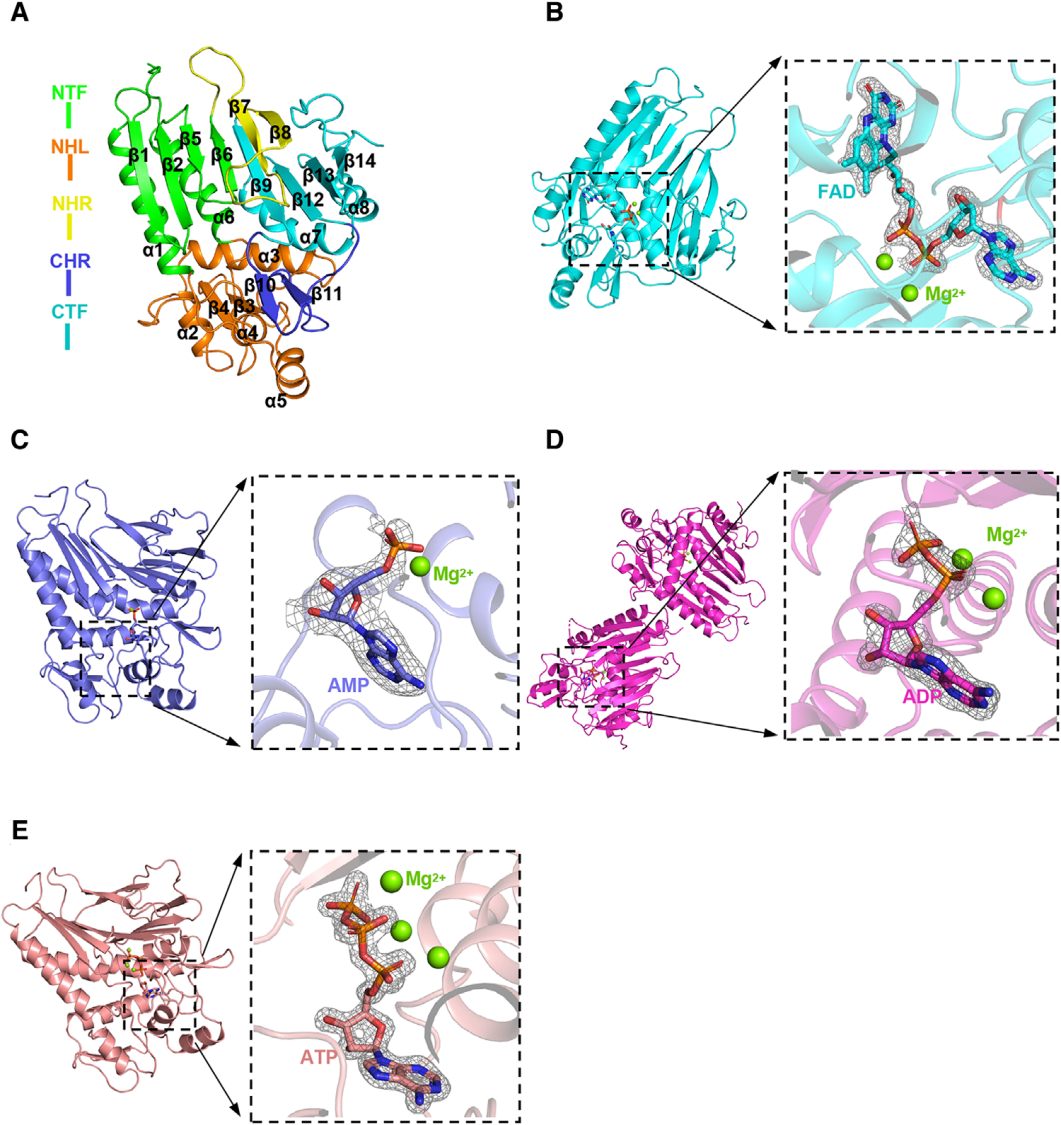
Overall structure of FmnB complexes. Proteins are represented by cartoons, different ligands are represented by corresponding sticks, and magnesium ions are represented by green spheres. A, Overall structure of apo‐FmnB. NTF/CTF, N/C‐terminal T‐shaped folds; NHR/CHR, N/C‐terminal β‐hairpin ring; NHL, N‐terminal α‐helices, and β‐lamellae. B–E, The overall structure of FmnB‐FAD, FmnB‐AMP, FmnB‐ADP, and FmnB‐ATP; the electron density around the corresponding ligand has been omitted. The 2Fo‐Fc omit map of the ligand (contoured at 1.8σ) is shown on the right

The structure of apo‐FmnB at 1.89 Å resolution was determined by X‐ray crystallography in the P2_1_2_1_2_1_ space group (Table S1). FmnB comprises three domains containing 8 α‐helices and 14 β‐strands (Figure 2A). The two fragments (residues 36–80 and 189–220) of the N‐terminal domain form a tunnel fold (N‐terminal T‐shaped fold, NTF) consisting of four antiparallel β‐strands and two antiparallel α‐helices. In addition, the two fragments (residues 251–260 and 287–357) of the C‐terminal domain form a structure similar to an NTF (C‐terminal T‐shaped fold, CTF). Four α‐helices and two β‐lamellae are formed between the two α‐helices of the NTF, named the NHL (N‐terminal α‐helices and β‐lamellae). A β‐hairpin ring (CHR) consisting of residues 261–285 is formed between the β9‐ and β12‐fold of the CTF. The CHR and NHL form an internal hydrophobic and externally polarized cavity inside FmnB (Figure S2). The CHR is located near the active center, which can regulate and stabilize the binding of the substrate FAD.

**FIGURE 3:**
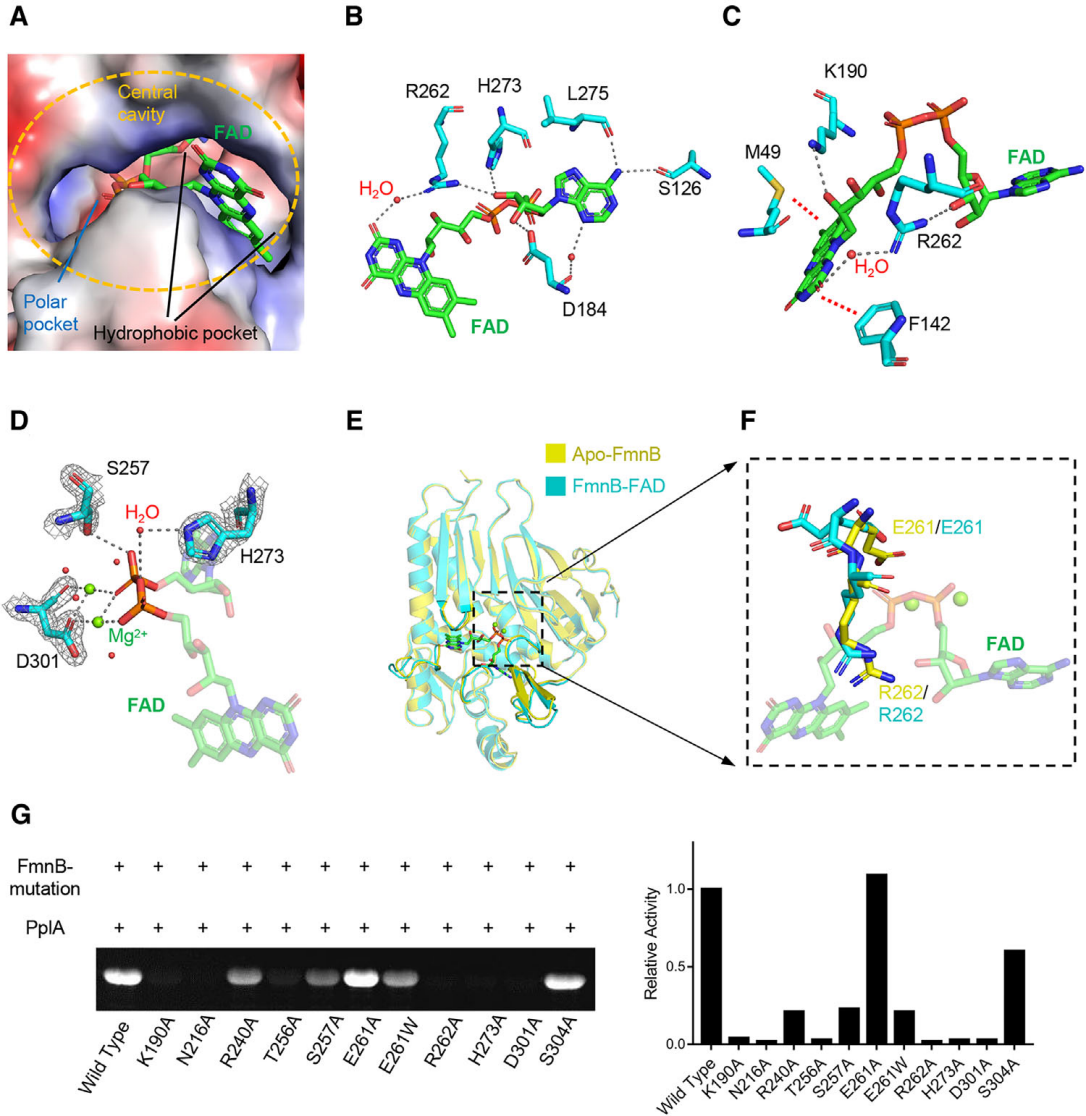
Structural basis of FmnB catalysis. A, Electrostatic surface representation of FmnB bound to FAD. FAD is shown as a stick representation within the active site. The large central cavity contains a polar pocket (red and blue) and a hydrophobic pocket (gray) for binding to the adenine and isoalloxazine rings of FAD, respectively. B, Interactions between FmnB and the adenine portion of FAD. C, Interactions between FmnB and the isoalloxazine rings of FAD. The red dashed lines indicate hydrophobic packing. Hydrogen bonds and salt bridges are shown in gray. D, Interactions between FmnB and the phosphate groups of FAD. E, Superposition of apo‐FmnB (yellow) and FmnB‐FAD (cyan). F, Conformational changes of the E261 and R262. G, Activity analysis of mutants of key residues of FmnB interacting with FAD

As defined by unbiased electron density, FAD in a bent conformation binds to the central cavity of FmnB with the diphosphate nearly perpendicular to the adenine (Figure 2B). The adenine of FAD is buried deep in the hydrophobic pocket inside the cavity, and the isoalloxazine ring is exposed outside the hydrophobic cavity. However, the two sides of the diphosphate inside the cavity are acidic amino acids and basic amino acids, which potentially fix the phosphate conformation via the pull–push effects of the opposite charges (Figures 3A and S3).

### 2.3 Recognition of FAD and Mg^2+^

Binding of FAD by FmnB occurs through a combination of extensive polar and hydrophobic interactions. The adenine of FAD forms hydrogen bonds with Ser126 and Leu275 of FmnB and establishes water‐mediated hydrogen bonds with the carbonyl group on the main chain of Asp184. Additionally, the sugar ring of FAD forms salt bridges with His273 and Arg262 and hydrogen bonds with the side chain of Asp184. Together, these interactions cause the ribitol of FAD to be completely embedded in the inner pockets of the cavity (Figures 3B and S3B). In contrast, fewer interactions occur between the isoalloxazine ring and FmnB. Instead, the isoalloxazine ring is sandwiched between Phe142 and Met49 through the hydrophobic interactions (Figures 3C and S3B). Remarkably, Arg262 not only forms a salt bridge with the sugar ring but also forms water‐mediated hydrogen bonds with isoalloxazine ring, which likely maintains the curved conformation of FmnB‐bound FAD (Figure 3B and C). In addition, FAD further forms hydrogen bonds with Met49 and a salt bridge with Lys190 to stabilize the spatial conformation of the isoalloxazine ring (Figure 3C). Collectively, these interactions further help the isoalloxazine ring of FAD become exposed in the solvent and better release FMN after FAD is hydrolyzed. Moreover, the diphosphate of FAD is inserted further into the FAD‐binding pocket, forming hydrogen bonds with Ser257 and water‐mediated hydrogen bonds with His273 and coordinating with Mg^2+^ through Asp301 (Figure 3D).

The electron density map of the FmnB‐FAD crystal structure shows additional electron density adjacent to the diphosphate group (Figure S4A). Two Mg^2+^ ions are present in the electron density model, and they are coordinated with the phosphate groups as well as water molecules. In addition to these interactions, one of the Mg^2+^ ions (Mg1) coordinates with Asp301, Ser304, and Ala187. The other Mg^2+^ (Mg2) coordinates with only Asp301. These Mg^2+^‐mediated interactions might stabilize the binding of FAD and facilitate hydrolysis of FAD by FmnB (Figure S4B). Indeed, substitutions of these amino acids by alanine greatly reduced or even abolished the enzymatic activity of FmnB (Figure 3G).

To verify the importance of the FAD‐interacting residues identified in the crystal structure, we generated several key FmnB variants and evaluated the enzymatic activity of these variant proteins (Figure 3G). Consistent with our structural observations, mutating Lys190, Arg262, and His273 to alanine abrogated the enzymatic activity. However, mutating Ser257 (S257A) only reduced the enzymatic activity. Specifically, the electron density map of residue Glu261 shows that Glu261 has two different conformational states (Figure S4C). To identify the conformational state of Glu261 that is most conducive to the enzymatic activity of FmnB, we tested the enzyme activity of two mutants, namely, E261A and E261W. The results showed that mutation of Glu261 to alanine enhanced the enzymatic activity, while mutation to tryptophan decreased the activity (Figure 3G), indicating that Glu261 affects the charge distribution near the phosphate group of FAD (Figure S3C).

### 2.4 Conformational changes of FmnB in inactive and active states

To further explore the catalytic mechanism of FmnB, we compared the structures of Apo‐FmnB and FmnB‐FAD. This result shows that the binding pocket of FmnB‐FAD is slightly larger. Moreover, residues 83–87 form a flexible loop, which may stabilize the spatial position of the isoalloxazine ring and make it easier to release FMN after FAD is hydrolyzed (Figure 3E). Obviously, large conformational changes occur in the CHR domain. However, only the catalytic residues Glu261 and Arg262 showed significant conformational changes in their side chains (Figure 3F). Glu261 adopted a rotamer conformation that removed it from the catalytic center through charge repulsion, and Arg262 no longer occupied the position of the sugar ring but formed a salt bridge with the sugar ring to stabilize the FAD conformation. Collectively, these results indicate an important role for the charge repulsion of Glu261 and the interaction of Arg262 in the binding of FAD by FmnB.

Structure‐based sequence alignments showed that FmnB shares 30% sequence identity with Gram‐negative bacterial sequences that have been reported^12^, ^13^, ^14^ (Figure S1). However, the structure of FmnB is obviously different from that of TpFmnB (RMSD: 1.49), PsFmnB (RMSD: 2.55), and VcFmnB (RMSD: 1.98) (Figure S5). Although these homologous proteins all adopt a similar tunnel fold, only Asp301, His273, and Ser257 at the catalytic centers were highly conserved, and the key binding site residues surrounding the FAD‐binding region were not conserved. As shown in the red dashed box, there are significant differences in their CHR and CTF domains, especially in PsFmnB. These results may provide an explanation for the different substrate specificity of these related enzymes. What's more, the sequence of FmnB is highly conserved in most of the homologs from Gram‐positive bacteria compared with those from Gram‐negative bacteria (Figure S6), indicating that a similar catalytic mechanism may be employed in Gram‐positive bacteria.

### 2.5 Structural basis of inhibition of FmnB activity by adenosine phosphates

**FIGURE 4:**
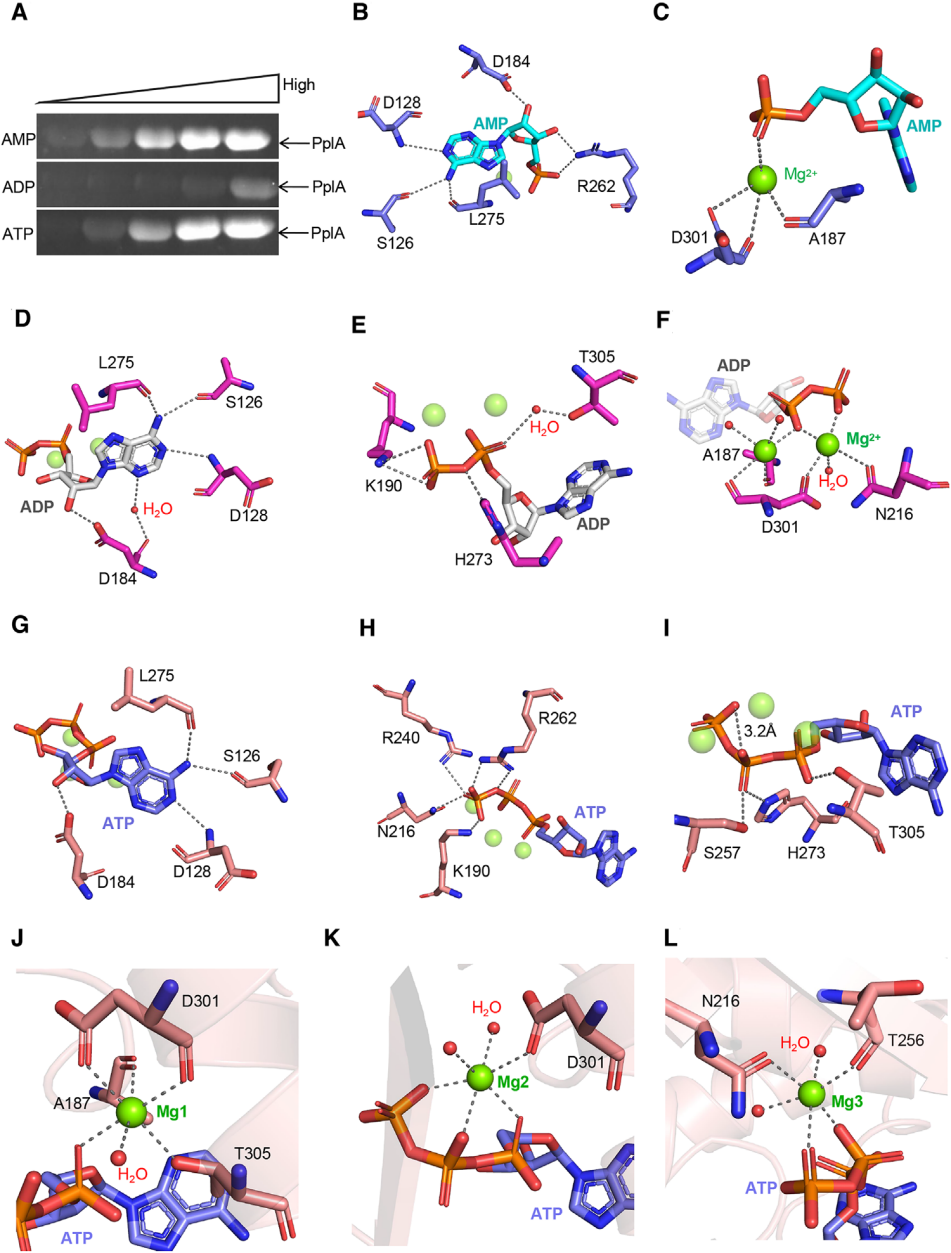
Recognition of AMP, ADP, ATP and Mg^2+^ ion. A, Effects of different inhibitors (AMP, ADP and ATP) on FmnB activity. The concentrations, from low to high, are as follows: 10 μM, 50 μM, 250 μM, 1 mM, 4 mM. B, Binding sites of AMP. C, The Mg^2+^ ion in the active site of FmnB‐AMP. D, E, Binding sites of ADP. F, The Mg^2+^ ion in the active site of FmnB‐ADP. G–I, Binding sites of ATP. J–L, The Mg^2+^ ion in the active site of FmnB‐ATP

As reported, AMP, the hydrolysis product of TpFmnB,^12^ could inhibit the activity of this enzyme, as well as the analogs ADP and ATP, whereas ADP exhibited the ability to promote VcFmnB^15^ activity in previous reports. However, in this study, ADP demonstrated a much stronger inhibitory ability against FmnB activity (Figure 4A). To understand the discrepancies and reveal the inhibitory mechanisms, we determined a set of structures of FmnB complexed with AMP, ADP, and ATP at high resolutions (Figure 2C‐E).

Based on structural observations, AMP, ADP, and ATP all bind to FmnB in a curved conformation. Similar to FAD, the phosphate group is almost perpendicular to the adenine (Figure 2C‐E). Additionally, their ribitols all interact with Asp128, Ser126, Leu275, and Asp184 of FmnB (Figure 4B, D, and G). These interactions cause their ribitol to be completely inserted into the hydrophobic pocket (Figures S7A, S8A, and S9A). In contrast, for FmnB‐AMP, the phosphate and sugar ring form salt bridges with Arg262 of FmnB to stabilize the bent conformation of AMP (Figure 4B). For FmnB‐ADP, the phosphate groups of ADP form salt bridges with Lys190 and His273 and a water‐mediated hydrogen bond with Thr305 (Figure 4E). For FmnB‐ATP, the γ‐phosphate group of ATP forms salt bridges with Lys190, Arg262, Asn216, and Arg240 (Figure 4H). In contrast, fewer interactions occur between the α‐ and β‐phosphate groups of ATP and FmnB. Notably, a hydrogen bond is established between the β‐phosphate group and γ‐phosphate group, which might be essential to stabilize the bent conformation of ATP (Figure 4I). Moreover, as observed on the electrostatic surface of inhibitor‐bound FmnB, the binding pocket of phosphate groups is polarized with positive and negative charges. AMP and ADP are encapsulated between acidic and basic amino acids, respectively, while ATP is inserted into positively polarized pockets. In other words, with the increase in the number of phosphate groups, the positive polarization of one side of the cavity gradually increases, while the negative polarization of the other side gradually decreases. Collectively, the results indicate that the phosphate groups of adenosine phosphates can affect the charge distribution of the active center and thus affect the catalytic activity of FmnB (Figures S7B, S8B, and S9B).

The electron density map of the inhibitor‐bound FmnB crystal structure shows one region of electron density adjacent to AMP, two adjacent to ADP, and three adjacent to ATP. A single Mg^2+^ ion was modeled in the electron density region near the AMP, and this ion coordinates with Asp301, Ala187, and the phosphate group (Figure 4C). Similarly, the Mg^2+^ ions near the ADP coordinate Asp301, Ala187, Asn216, and the phosphate groups as well as water molecules (Figure 4F). The Mg^2+^ ions near the ATP coordinate Asp301, Ala187, Thr305, Asn216, Thr256, and the phosphate groups as well as water molecules (Figure 4J‐L). Collectively, these Mg^2+^‐mediated interactions might stabilize the local conformation of AMP, ADP, and ATP.

### 2.6 Identification of specific inhibition of FmnB activity

**FIGURE 5:**
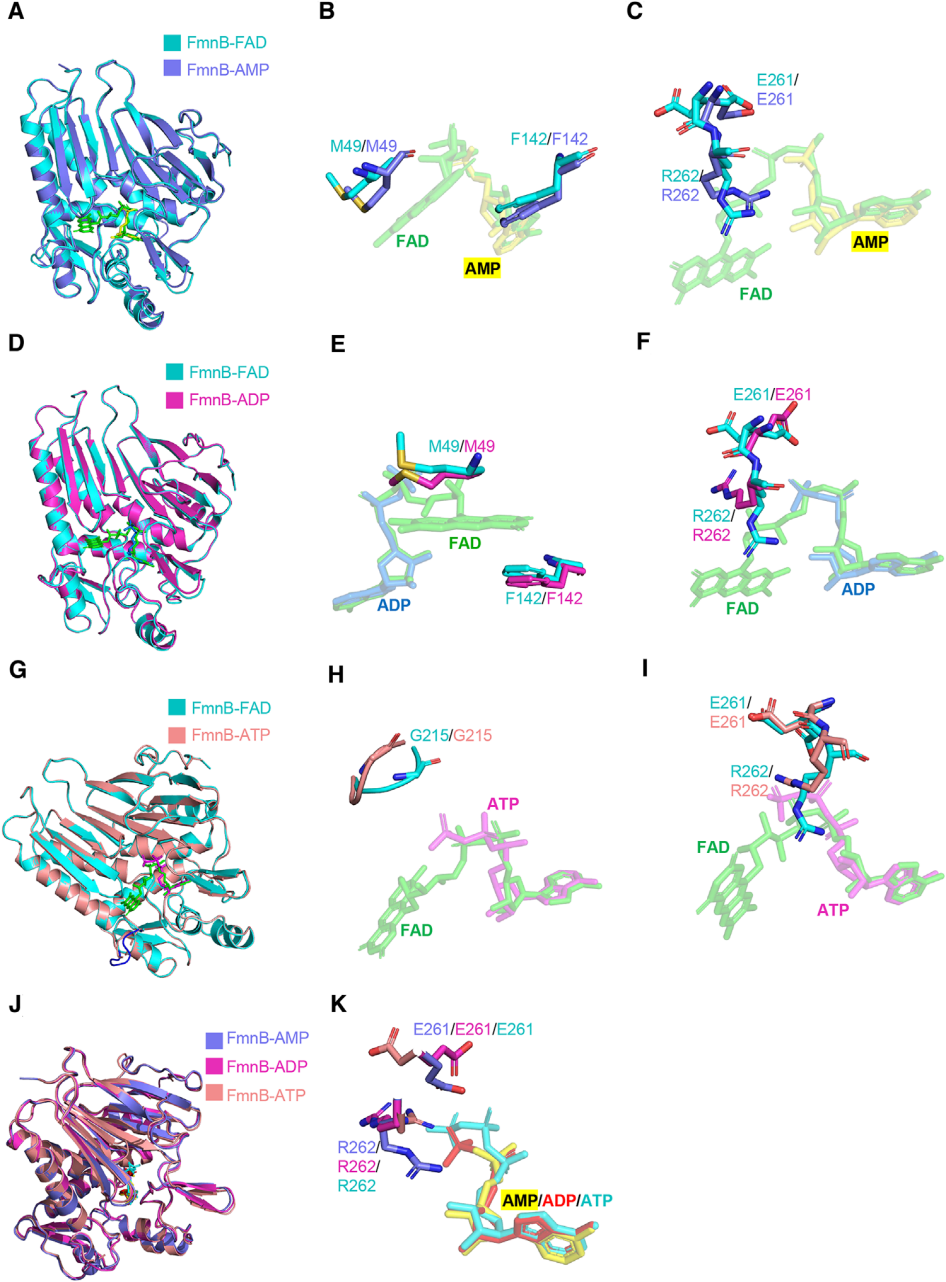
Structural basis of FmnB inhibition. A–C, Superposition of FmnB‐FAD and FmnB‐AMP. FAD is shown as green sticks. AMP is shown as yellow sticks. D–F, Superposition of FmnB‐FAD and FmnB‐ADP. ADP is shown as aquamarine sticks. G–I, Superposition of FmnB‐FAD and FmnB‐ATP. ATP is shown as magenta sticks. Residues 82–89 are shown in the blue loop. J–K, Superposition of E261 and R262 in FmnB‐AMP (slate), FmnB‐ADP (magenta) and FmnB‐ATP (salmon)

To reveal the effect of adenosine phosphates on the conformation of FmnB, we set up a series of structural comparisons of FmnB in complex with the substrate and different inhibitors. The structural differences between the FmnB‐FAD and FmnB‐AMP complexes primarily lie in the binding of the isoalloxazine ring and phosphate groups of FmnB (Figure 5A). The binding pocket of FAD is slightly larger than AMP. However, Met49 and Phe142 of the stabilizing isoalloxazine ring show no obvious conformational change (Figure 5B); in contrast, Glu261 and Arg262 exhibit many conformational changes (Figure 5C). Arg262 forms salt bridges with the sugar ring and phosphate group of AMP to stabilize the AMP conformation. Glu261 is closer to the center of the cavity because of the single phosphate group of AMP (Figures 4B and 5C). Furthermore, while FmnB‐ADP is similar to FmnB‐AMP, the binding pocket for ADP is smaller, and there are no significant conformational changes in Met49 and Phe142 (Figure 5D and E). In particular, Arg262 is more distant (4.3 Å) from the β‐phosphate group of ADP, possibly due to spatial repulsion between Arg262 and the phosphate group (Figures 5F and S10B). Finally, for FmnB‐ATP, the distance between Gly215 and the γ‐phosphate group of ATP is significantly increased due to steric limitation (Figure 5H). Moreover, residues 83–86 of ATP‐bound FmnB are lost due to poor electron density (Figure 5G). Notably, Glu261 is at a greater distance from ATP due to greater charge repulsion, similar to that in FmnB‐FAD, and Arg262 is adjacent to the β‐ and γ‐phosphate groups of ATP (Figure 5I). Collectively, these results indicate that adenosine phosphates significantly affect the conformation of Arg262 in addition to occupying the position of FAD (Figure 5J and K).

To verify the conformational change in Arg262, we generated a mutant with alanine replacing Arg262 of FmnB and evaluated the ability of this mutant to bind to various inhibitors (Figure S11). Consistent with our structural observations, substituting Arg262 with alanine resulted in no obvious conformational change. Unexpectedly, substitution of Arg262 with alanine resulted in an increased affinity for FmnB and ATP, indicating that the steric hindrance of Arg262 is not conducive to ATP binding. Since the affinity between AMP and FmnB may be outside of our detection range, we were unable to accurately measure the change in the affinity between AMP and FmnB. However, according to the clear electron density of the Arg262 side chain of FmnB‐AMP (Figure S10A), we believe that replacement of Arg262 will result in a significant decrease in the ability of FmnB to bind AMP. Collectively, these results show that the adenosine phosphates competitively inhibited the activity of FmnB mainly by affecting the conformation of Arg262.

Structure‐based sequence alignments showed that Glu261 and Arg262 of FmnB are relatively well conserved with TpFmnB.^12^ However, these two residues are not structurally conserved (Figure S12). ADP in TpFmnB interacts with Glu244 and Arg245, and ADP and AMP do not affect the conformation of Arg245 in TpFmnB. These differences suggest that the conformational changes caused by ADP are specific in *L. monocytogenes*.

## 3 DISCUSSION

Here, the structural and biochemical characteristics of the flavin transferase FmnB were reported, which is involved in EET in *L. monocytogenes*. Enzymatic activity assays demonstrated that FmnB is a Mg^2+^‐based FMN transferase. As our experimental results showed, Mn^2+^, Ni^2+^, Fe^2+^, and Co^2+^ have similar effects on the catalytic activity as Mg^2+^ (Figure 1C). This may be because they have similar physical properties, such as coordination patterns and atomic radii.^17^, ^18^ However, Ca^2+^, Cu^2+^, and Zn^2+^ have weak activation effects on FmnB, which may be because Cu^2+^ and Zn^2+^ have tetrahedral coordination in metalloproteins and are not ideal catalysts for metal ions to stabilize the binding of substrate FAD. In addition, Ca^2+^ occupies more space because of its larger atomic radius. Monovalent metal ions, such as K^+^, Li^+^, and Na^+^, do not seem to act as cofactors to activate the catalytic activity of the enzyme. It may be due to the small size of these atoms and their difficulty in forming coordination with residues of FmnB.

**FIGURE 6:**
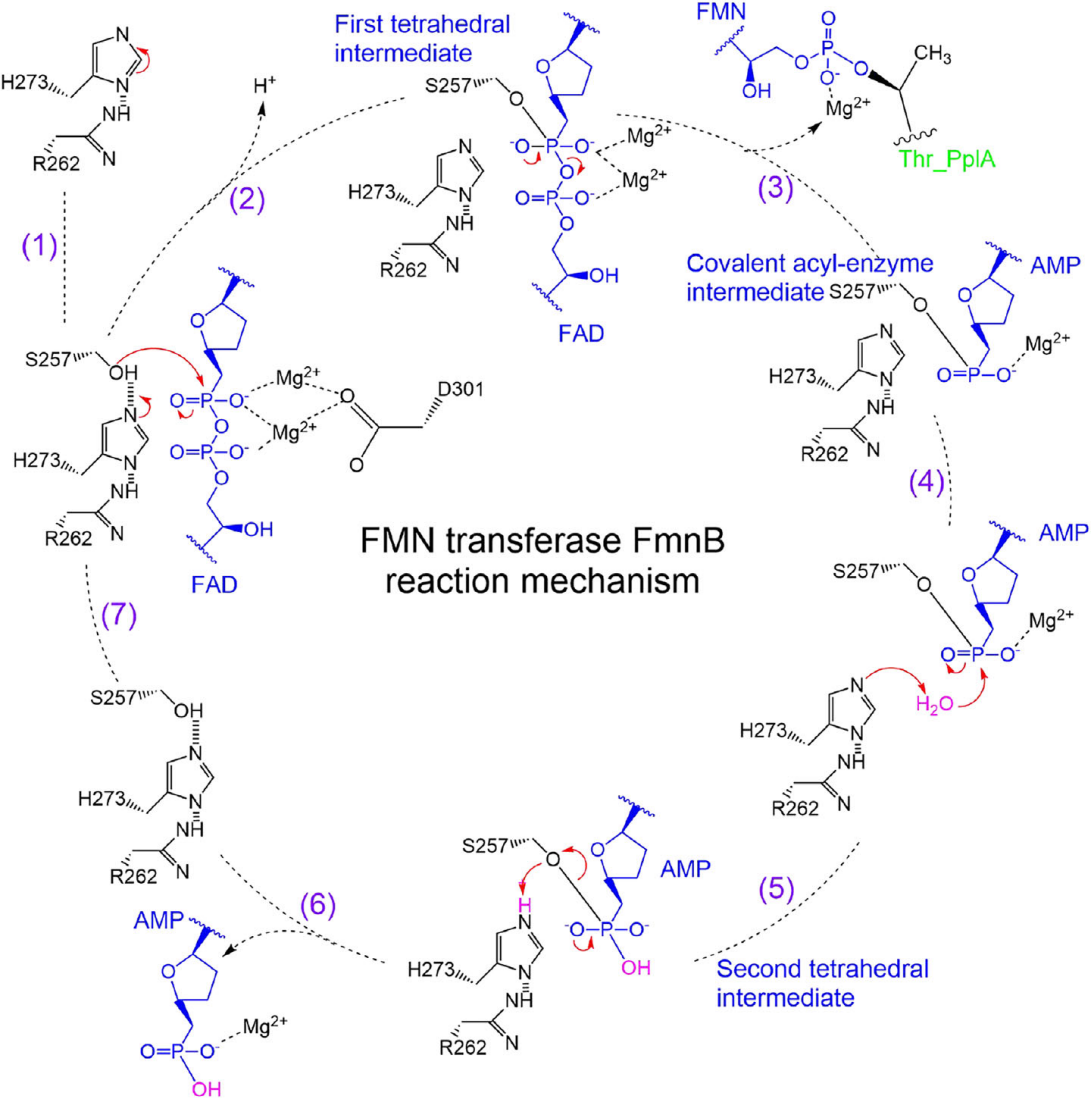
Reaction mechanism for hydrolysis of FAD by FmnB. The proposed FMN transferase reaction mechanism. The schematic depicts a triplet (D301‐H273‐S257) catalytic mechanism. FAD is the substrate of FmnB, and AMP and FMN are the products. The red arrows indicate electron transfers

Based on the high‐resolution X‐ray crystallographic structures and biochemical data, a reaction mechanism of FmnB‐catalyzed flavin transfer is proposed (Figure 6). The combination of Ser126, Asp184, and Leu275 creates a deep hydrophobic pocket inside FmnB, and Met49 and Phe142 form a hydrophobic cavity on the surface of FmnB, which greatly improves the substrate specificity (Figures 3A and B and S3B). The hydrolysis of FAD by FmnB is regulated by a typical triplet (Asp301‐His273‐Ser257). When FAD binds to FmnB, Arg262 increases the pKa of the imidazolium nitrogen of His273 from 7 to approximately 12 by hydrogen bonding with His273, resulting in His273 becoming a strong generalized base. Although nucleophilic residues used for covalent catalysis are generally acid‐base‐nucleophilic triplets, the shorter hydrogen bond (2.3 Å) between arginine and histidine removes the barrier to proton transfer, allowing protons to be shared in a broad single‐potential well (Figure S13A). It has been reported that low potential hydrogen bonding (<2.6 Å) occurs in the double proton transfer mechanism.^19^, ^20^, ^21^, ^22^, ^23^, ^24^ Although the acidic amino acid Glu261 is adjacent to Arg262, charge repulsion between Glu261 and the phosphate group prevents this residue from participating in proton transfer with His273. A similar phenomenon is observed in the cytomegalovirus protease^25^, ^26^ of the S21 family, which can use histidine as both an acid and a base in the triplet to form a serine‐histidine‐histidine (S‐H‐H) triplet.^27^ This explains why FmnB is more active at pH 10, as it easier for arginine to be ionized at this pH. Subsequently, His273 acts as a general base, removing a proton from Ser257 through a water molecule. Ser257 acts as a nucleophile to attack the phosphorus of the phosphorus‐oxygen bond and force it to accept electrons. Ser257 and Thr256 form hydrogen bonds via water molecules to stabilize the deprotonated Ser257 (Figure S13B). Because the p orbitals of oxygen and the d orbitals of phosphorus do not exactly match,^28^, ^29^ both phosphorus and oxygen, which can form only a double bond, have extra vacant orbitals that can hold more electrons, which allows the constant accumulation of electrons on oxygen to keep the substrate FAD in an active state. Meanwhile, Asp301 stabilizes the oxygen in the transition state through the coordination of magnesium ions, while Asn216 stabilizes Asp301 through a salt bridge with Asp301 (Figures 3D and S13C). This action is similar to the formation of oxyanion holes in the triplet catalysis of subtilisin, which is stabilized by Asn, Thr, and the backbone NH of Ser.^30^ Consistent with our enzymatic activity assays, substituting these residues with alanine resulted in significant or complete loss of enzymatic activity (Figure 3G). Notably, substituting Ser257 with alanine partially reduced the enzymatic activity. It is likely that the water molecule gives a proton to His273, and the remaining OH^−^ attacks the phosphorus‐oxygen bond to activate the hydrolysis of FAD. It has been reported that serine is dispensable in the catalytic triad.^31^ It has also been reported that hydrolytic water is involved in the mechanism of serine protease catalysis, requiring the formation of a hydrogen bond with His and an angle of attack of approximately 109°.^32^ This angle of attack is 108.1° for the water molecules near His273 during the catalytic process of FmnB (Figure S13D). This is probably why Ser257 is not indispensable. Finally, the hydrolysis product FMN of FmnB covalently interacts with Thr at residues 143 and 265 of PplA (Figure 6). In this process, threonine can also be replaced by serine. Since crystals of PplA are difficult to obtain, the specific electron transfer process through PplA remains to be studied.

Furthermore, it is evident that the common feature of AMP, ADP, and ATP in the inhibition of FmnB activity is that they all occupy the binding site of the substrate FAD. Moreover, based on the above discussion, Arg262 is required to stabilize FAD and activate nucleophilic attack, and Glu261 exhibits charge repulsion with the phosphate groups of FAD. Combined with the conformation of Glu261 and Arg262 in AMP‐, ADP‐, and ATP‐bound FmnB, the presence of AMP, ADP, and ATP redistributes the charge in the phosphate‐binding pockets. Because the phosphate‐binding pocket of FAD can be positively polarized or negatively polarized (Figure S3A), the presence of Glu261 affects the distribution of the positive charge (Figure S3C). Compared with the triphosphate group of ATP, the phosphate groups of AMP and ADP are not enough to cause charge repulsion with Glu261 to drive Glu261 away (Figures S7B and S8B). However, the larger negative charge of ATP triphosphate can repel the charge of Glu261 (Figure S9C). Collectively, these charge repulsions result in a significant decrease in ATP inhibition. Moreover, considering the effect of Arg262, only the presence of ADP causes Arg262 to move away from the center of the cavity. Notably, Arg262 cannot move away from the binding pocket of ATP, possibly because the triphosphate groups are completely inserted into the positively charged pocket. However, the presence of Arg262 is not conducive to the curved conformation of the triphosphate group, resulting in a significant decrease in the affinity between ATP and FmnB. In summary, these charge repulsion and space constraints result in the strongest inhibition of FmnB enzyme activity by ADP compared with that by AMP and ATP. An enzyme that balances rapidly between inactive and active conformations would rather have fewer conformational changes.^33^ These data suggest that a small group of structural elements control the specificity of flavin transferase. Such cases of fine regulation also occur in substrate catalysis by chymotrypsin‐like serine proteases.^34^, ^35^, ^36^, ^37^

Based on sequence alignment, these key residues were highly conserved in Gram‐positive bacteria. It is possible that the EET mechanism does not require complex assembly proteins that are dependent on polyheme and that the single‐membrane structure of Gram‐positive cells has enabled most Gram‐positive bacteria to evolve similar mechanisms. Therefore, the catalytic and inhibitory mechanisms of FmnB provide us with a new understanding of the flavin transferase in Gram‐positive bacteria, as well as a deeper understanding of the role of this enzyme in the EET mechanism.

## 4 CONCLUSION

The Mg^2+^‐dependent flavin transferase FmnB catalyzes and transfers flavin cofactors from the respiratory system of a variety of Gram‐positive bacteria. Therefore, it plays a key physiological role in the survival and pathogenicity of these Gram‐positive pathogens. In our work, we have demonstrated that FmnB follows a typical triplet (Asp301‐Ser257‐His273) catalytic mechanism and that residues Arg262 and Glu261 are critical for FmnB activity by structural and activity inhibition experiments. These results provide us with a deeper understanding of the flavin transferase family and the interplay between FmnB and the respiratory system of these pathogens.

## 5 MATERIALS AND METHODS

### 5.1 Construction of recombinant plasmid

Codons encoding FmnB and PplA proteins were linked to the NdeI and XhoI sites of the PET‐15B expression plasmid (Novagen, USA) and optimized according to the *Escherichia* coli expression system. These genes were derived from *L. monocytogenes*, eliminating the lead sequence. A 6× His tag was added to the N‐terminus of both proteins, and a termination codon was added to the C‐terminus. The three recombinant plasmids were transformed into *E. coli* DH5α cells and sequenced (Tsingke, China). To express these two proteins, the relevant plasmids were transferred into *E. coli* BL21 (DE3) cells.

### 5.2 Protein expression and purification

*E. coli* BL21 cells containing PplA or FmnB expressing plasmid were cultured in Luria broth (LB) medium at 37° supplemented with 0.1 g/L ampicillin or 0.05 g/L kanamycin until the optical density (OD_600_) at 600 nm reached 0.6. Next, the isopropyl β‐D‐thiogalactoside (IPTG) was added to LB medium with a final concentration of 0.2 mM and induced at 16°C for 16 h. FmnB with methionine replaced by selenomethionine (SE‐Met) was grown at 37°C in M9 medium containing 0.1 g/L ampicillin, 3 g/L KH2PO4, 0.5 g/L NaCl, 6 g/L Na2HPO4, 1 g/L NH4Cl, 0.4% glucose). After OD_600_ reached 0.6, the medium was slowly added NaOH with a final concentration of 45 mM and cooled to 25°C by shaking. Then, the medium was added with different amino acids (0.05 g/L L‐valine, 0.05 g/L L‐leucine, 0.05 g/L L‐isoleucine, 0.1 g/L L‐threonine, 0.1 g/L L‐phenylalanine, 0.1 g/L L‐lysine, and 0.1 g/L L‐Se‐methionine) and cooled to 16°C after shaking for 30 min. After 0.2 mM IPTG was added, the protein was overexpressed at 16°C for about 16 h.

Cell pellets were collected by centrifugation at 3800 rpm for 15 min and suspended in lysis buffer contain 300 mM NaCl, 25 mM Tris/HCl 8.0, and 1 mM phenylmethylsulfonyl fluorid (PMSF) and cleaved by ultrasound. The supernatant containing the target protein was streamed through a Ni‐NTA column (NI‐NTA; GE Healthcare, UK) prebalanced suspension buffer. The resin was then eluted with different concentrations of imidazole (10, 20, 30 mM). Finally, the crude target protein was preliminarily obtained by lysis solution added 300 mM imidazole. Subsequently, the eluted protein is further purified by anion exchange column (Source Q; GE Healthcare) and size‐exclusion chromatography (Superdex 200 10/300; GE Healthcare) prebalanced by buffer containing 15 mM Tris/HCl (pH 8.0) and 150 mM NaCl. The collected proteins were verified by sodium dodecyl sulfate‐polyacrylamide gel electrophoresis (SDS‐PAGE). The highest concentration of protein was used for crystal growth.

### 5.3 Crystallization and data collection

The protein (about 10 mg/ml) prepared in advance is used for crystal growth. Protein crystals were preliminarily screened in Index (Hampton Research, USA), PEG/ION (Hampton Research, USA) and WIZard3/4 (Hampton Research, USA) kits by the sitting‐drop vapor‐diffusion method^38^ at 16°C. Each well was added to a mixture containing 1 μl of protein solution and an equivalent amount of reservoir solution.

The apo‐FmnB crystal was obtained under a variety of conditions. The crystal of the resolved structure is obtained in the solution containing 0.2 M Potassium sodium tartrate tetrahydrate, 20% w/v polyethylene glycol 3350. The crystal reaches a more rational crystal shape after 3 days. The crystals are frozen in liquid nitrogen with 15% glycerol as cryoprotectant and quickly frozen before X‐ray diffraction. The crystals of FmnB complexed with FAD, AMP, ADP, and ATP were obtained by incubation of FmnB and the corresponding ligand, and a small amount of ligand was added to the antifreeze solution before harvesting the crystals.

X‐ray diffraction data were collected by a Pilatus3 6 M detector at 100 K. All the datasets were collected at beamline BL18U1 of the Synchrotron Radiation Facility in Shanghai.^39^ A total of 360 images were obtained at a 0.25 s exposure at a distance of 400 mm from the crystal to the detector, covering a total rotation range of 360 using 1.0 oscillations. The collected data were indexed, integrated, and scaled using the HKL3000 software suite.^40^ Using the anomalous data of SE‐Met crystal, the crystal structure of FmnB‐FAD was solved by using the single wavelength anomalous dispersion (SAD) phase method. The final model was manually built in Coot and optimized in PHENIX. The final models were validated by MolProbity and deposited in the Protein Data Bank (see Table S1).

### 5.4 Site‐directed mutagenesis

All single mutations were generated according to the reported protocol.^41^ Mutations were amplified and sequenced using heat‐activated high‐fidelity DNA polymerase PCR (Qingdao, China). These mutants are expressed and purified in the same way as wild‐type proteins.

### 5.5 FmnB activity measurement

FmnB catalyzes the transfer of flavin to PplA and takes FAD as a substrate to produce AMP. The yellow color of PplA can be observed due to FMN fluorescence in an SDS gel under UV irradiation, which provides a relatively simple and reliable method to track the physiological activity of FmnB.^42^, ^43^ The purified protein was incubated with 5 mM Mg^2+^ and FAD in reaction buffer containing 15 mM HEPES 7.0 and 150 mM NaCl. Briefly, the standard 100 μl reaction containing 5 μM FmnB and 5 mM Mg^2+^ was incubated for 15 min at 16°C. Then, 50 μM FAD and 100 μM PplA was added. The reaction was performed at 16°C for 20 min and stopped by the adding of 5× SDS‐PAGE loading buffer. The samples were run in SDS‐PAGE gel, which was then exposed to UV light before staining. The fluorescence intensity in the gel images was analyzed using ImageJ.^44^

### 5.6 FmnB pH‐dependent assay

To inspect the pH dependence of wild‐type FmnB, the assay was carried out in buffer containing 15 mM HEPES, 15 mM Tris, 150 mM NaCl, and 5 mM MgCl_2_ at a series of pH values (5.0‐10.0). 100 μM FAD, 5 mM Mg^2+^, and 50 μM PplA (100 μM) were mixed in different pH buffer, and finally 5 μM FmnB was added to react at room temperature for 20 min. The reactions were stopped by adding 5× SDS‐PAGE loading buffer. The product was determined as described above.

### 5.7 Substrate specificity

To investigate the substrate specificity of FmnB, the reaction was set up with 5 μM FmnB, 100 μM FAD, 15 mM Mg^2+^, and 50 μM PplA in reaction buffer. Different metal ions other than Mg^2+^ were added at the same concentration. To test the effect of the products of FmnB on activity, different concentrations of AMP, ADP, and ATP were added to the solution above. All the reactions were performed for 30 min at 16°C and stopped by adding 5× SDS‐PAGE loading buffer.

### 5.8 Microscale thermophoresis (MST)

To investigate the ability of FmnB to bind to AMP, ADP, and ATP, an affinity assay was performed using MST (Monolith NT.115). The protein was diluted in 15 mM HEPES 7.5 and 150 mM NaCl for affinity measurement. The micromolecule was subjected to a 1:1 dilution in PCR tubes. The protein was added to a final concentration in the range of 50–200 nM. Due to the weak interaction between AMP and FmnB, the AMP concentration required for binding with FmnB was higher than of the ADP and ATP concentrations. The samples were run in standard capillaries with a blue filter on a Monolith NT.115 Capillary with MST power 60 or 80. The equation used to analyze the equation are described below.

$$C_{hot}/\;C_{cold}=e^{-S_{T}\Delta T},$$

$$\Delta \;F_{norm}=F_{hot}\;/F_{cold}\cdot 1000,$$

where *S_T_* is the Soret coefficient, *C_hot_* and *C_cold_* represent the concentration in the heated and nonheated regions of the capillary. Δ*T* is the change in temperature. The experiments were carried out in triplicate using the same batch of protein and small molecules.

## CONFLICT OF INTEREST

The authors declare that they have no conflicts of interest.

## ETHICS APPROVAL

No ethical issues are involved in this study.

## AUTHOR CONTRIBUTIONS

Wei Cheng designed the research; Yanhui Zheng carried out the biochemical and structural studies, participated in the data analyses, and drafted the manuscript. Chao Dou and Weizhu Yan participated in the structure determination and biochemical experiments. Weizhu Yan, Dan Zhou, Ying Jin, Yunying Chen, Xiaotao Zeng and Lulu Yang purified the proteins and grew the crystals. Wei Cheng conceived the study, participated in the experimental design and data analysis, and modified the manuscript.

## Supporting information

Supporting information

## Data Availability

The coordinates and structure factors of apo‐FmnB and the complexes of FmnB with FAD, AMP, ADP and ATP have been deposited at the RCSB PDB with the assigned PDB codes 7F39, 7ESA, 7ESC, 7F2U and 7ESB, respectively.
