# Supplementary material for: Structural insights into the catalytic and inhibitory mechanisms of the flavin transferase FmnB in Listeria monocytogenes
Source: MedComm (2020). 2022 Jan 10;3(1):e99. doi: 10.1002/mco2.99 (PMC8906456; doi:10.1002/mco2.99)
Supplement: Supplementary file 1 — Supporting information [file MCO2-3-e99-s001.pdf]

Structural insights into the catalytic and inhibitory mechanisms of the flavin transferase FmnB in *Listeria monocytogenes*

Yanhui Zheng[1†], Weizhu Yan[1†], Chao Dou[1], Dan Zhou[1], Yunying Chen[1], Ying Jin[1], Lulu Yang[1], Xiaotao Zeng[1], Wei Cheng[1*]

[1]Division of Respiratory and Critical Care Medicine, Respiratory Infection and Intervention Laboratory of Frontiers Science Center for Disease-Related Molecular Network, State Key Laboratory of Biotherapy, West China Hospital of Sichuan University, Chengdu 610041, China.

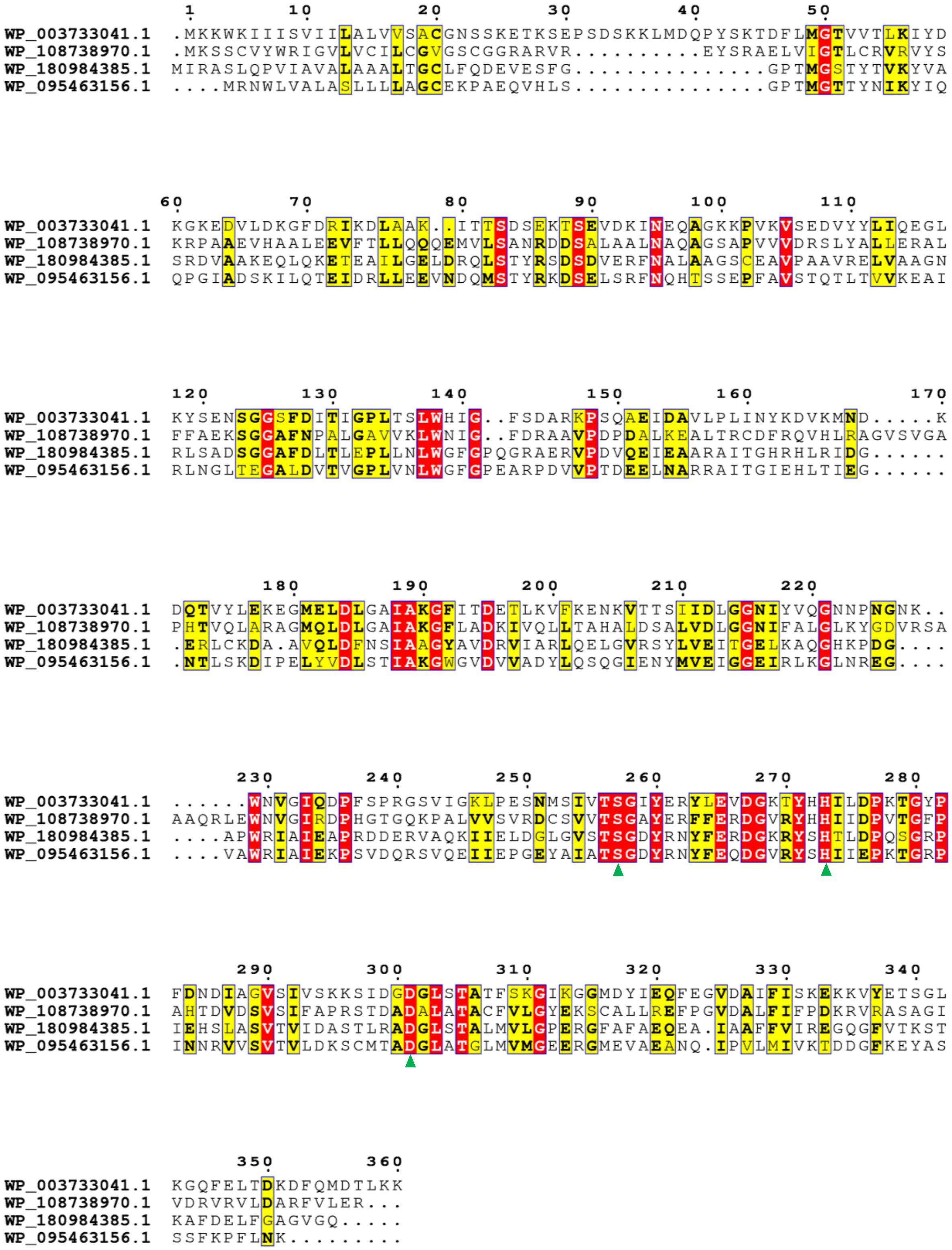

Figure S1. Sequence alignment of FmnB homologs in gram-negative bacteria. Amino acid sequence alignment of WP_003733041.1 (FmnB, *Listeria monocytogenes*), WP_108738970.1 (TpFmnB, *Treponema pallidum*), WP_180984385.1 (PsFmnB, *Pseudomonas stutzeri*), and WP_095463156.1 (VcFmnB, *Vibrio cholerae*). Highly conserved residues in the catalytic site are indicated by green triangles.

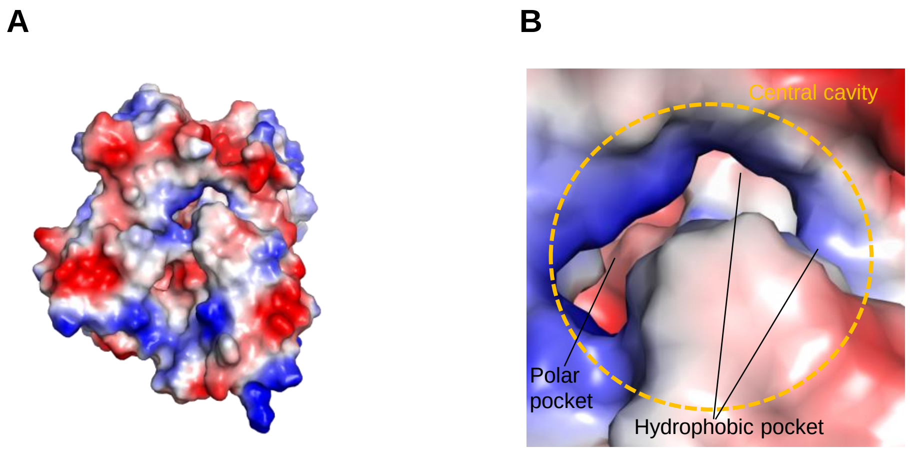

Figure S2. Electrostatic surface representation of FmnB-Apo. The large central cavity contains a polar pocket (red and blue) and a hydrophobic pocket (gray).The electrostatic potential was computed using the APBS tools in PyMol (http://www.pymol.org/).

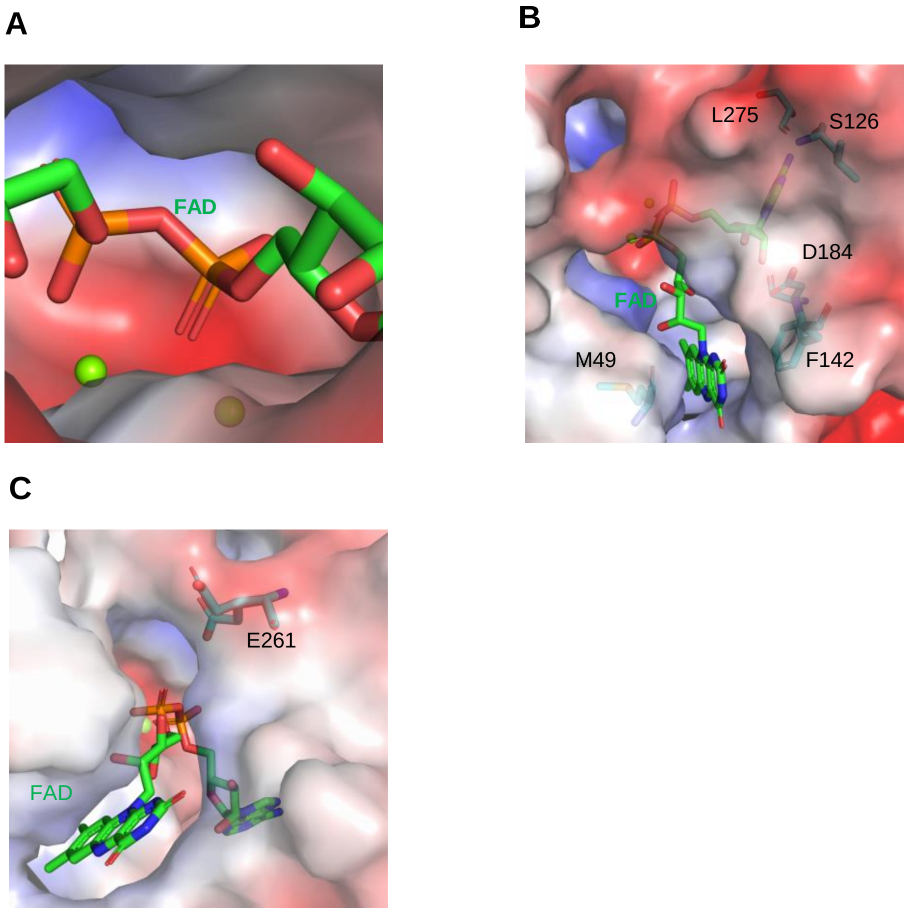

Figure S3. Electrostatic surface representation of FmnB-FAD. A, The binding pockets of the phosphate groups are polarized by positive (blue) and negative (red) charges. **B,** The adenine of FAD is buried deep in the hydrophobic pocket composed of S126, D184 and L275. The isoalloxazine rings of FAD are exposed outside the hydrophobic cavity composed of M49 and F142. **C,** Electrostatic potential at the surface of E261 in FmnB-FAD. The electrostatic potential was computed using the APBS tools in PyMol (http://www.pymol.org/).

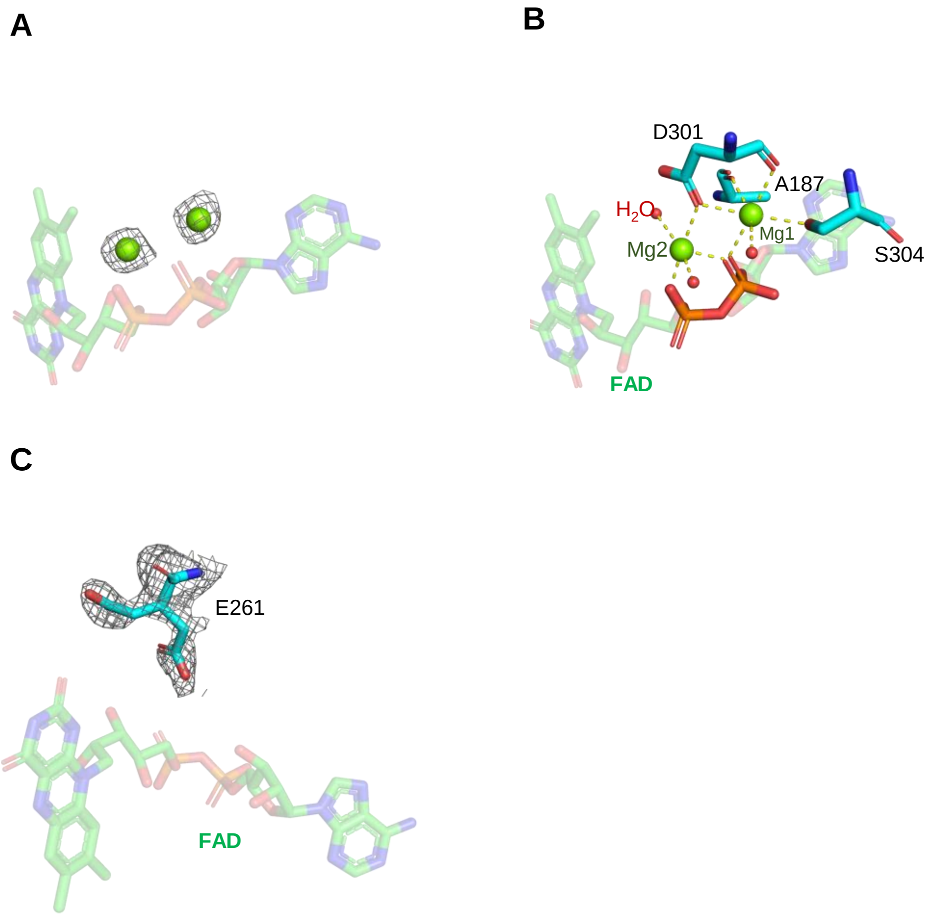

Figure S4. Electron density maps of the structure of FmnB bound to Mg^{2+} ions and E261. A, 2Fo-Fc omit map of Mg^{2+} (in green) contoured at 2.2σ is shown in gray. **B,** Coordination of Mg^{2+} in FmnB-FAD. **C,** 2Fo-Fc omit map of E261 (in cyan) contoured at 1.0σ is shown in gray.

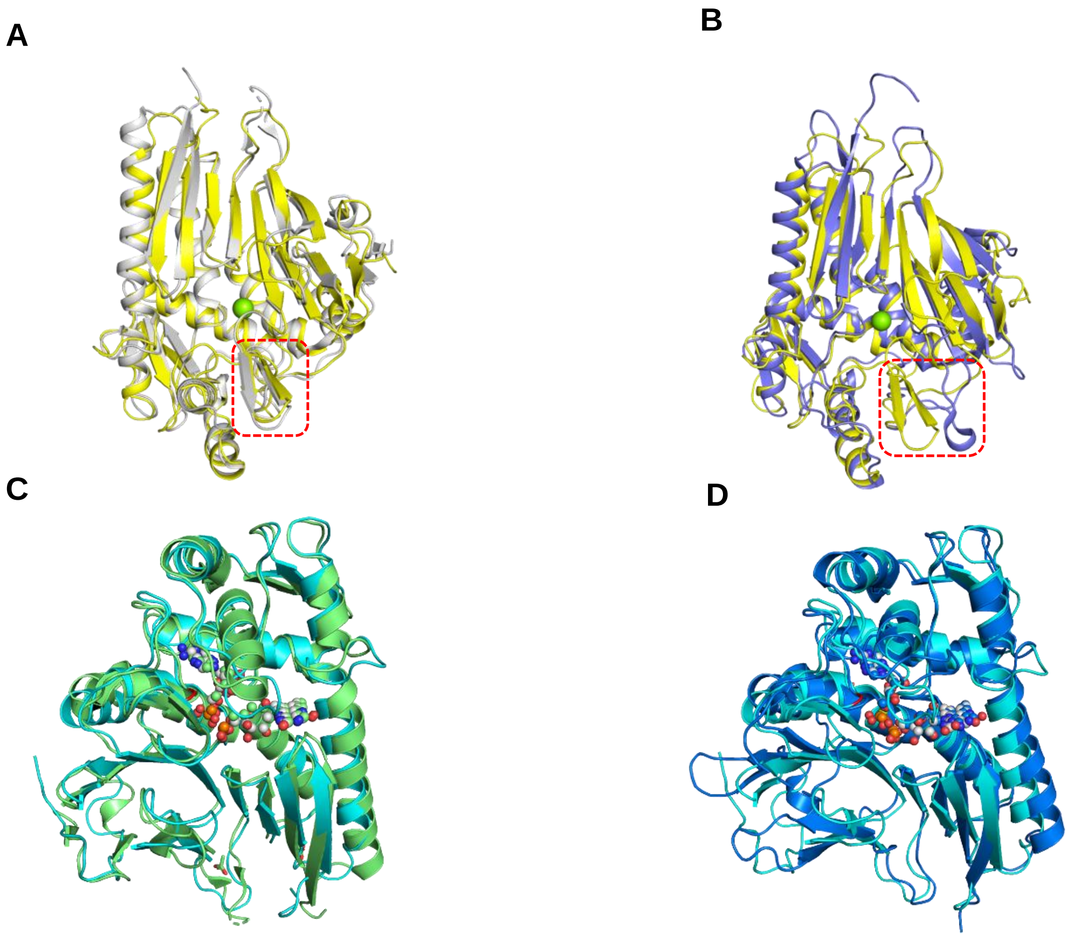

Figure S5. Structural alignment of FmnB homologs. A and B, Superposition of apo-FmnB (yellow), apo-TpFmnB (gray; PDB: 4XFU) and apo-PsFmnB (slate; PDB: 5MGY). Magnesium ions are represented by green spheres. **C and D,** Superposition of FmnB-FAD (cyan), TpFmnB-FAD (green; PDB: 4IFX) and VcFmnB-FAD (aquamarine ; PDB: 6NXI). The FAD ligand is represented by spheres.

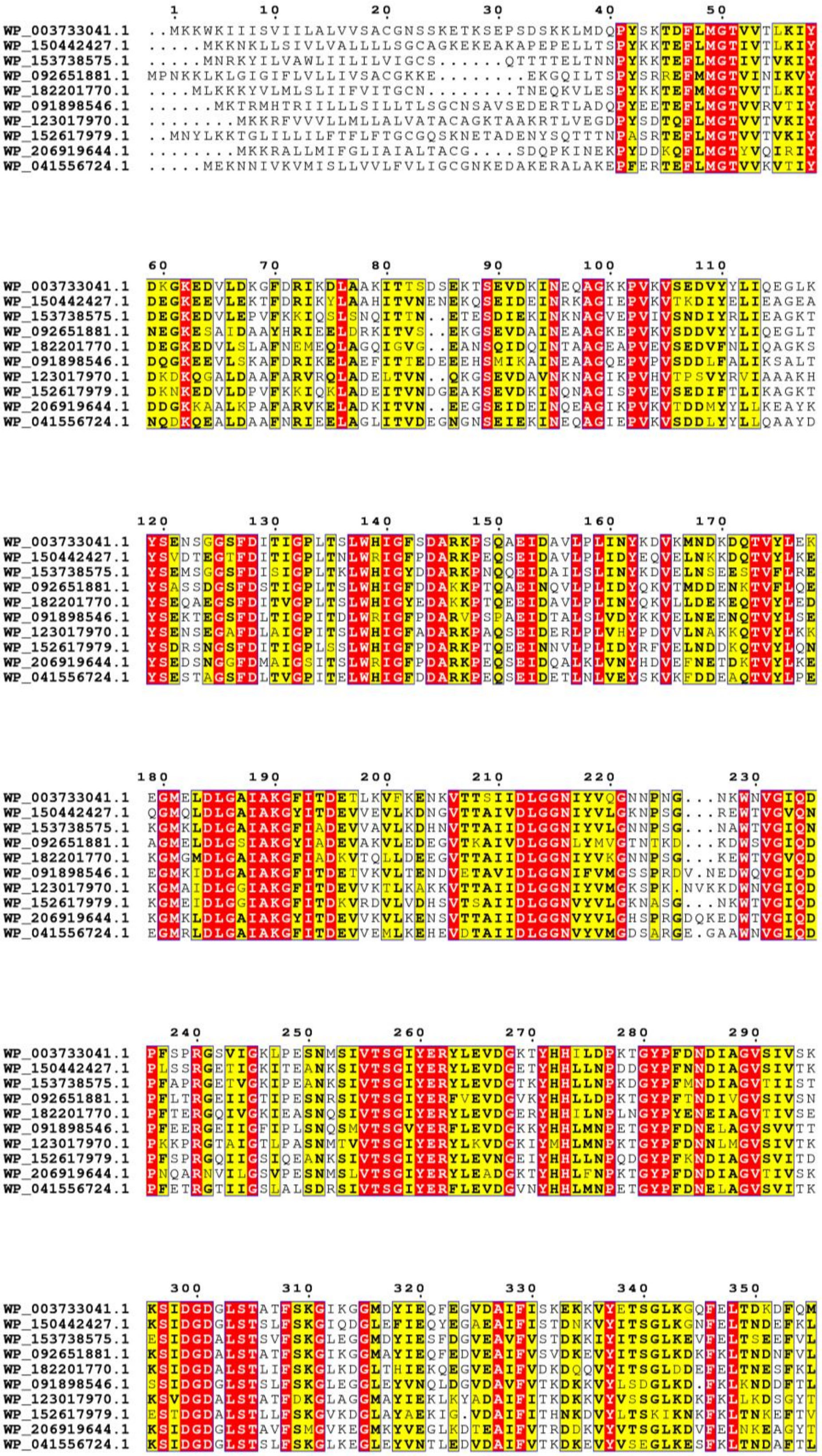

Figure S6. Sequence alignment of FmnB homologs in gram-positive bacteria. Amino acid sequence alignment of WP_003733041.1(FmnB, Listeria monocytogenes), WP_150442427.1 (Bacillus endozanthoxylicus), WP_153738575.1 (Aquibacillus halophilus), WP_092651881.1 (Isobaculum melis), WP_182201770.1 (Paraliobacillus sp. G6-18), WP_091898546.1 (Marinilactibacillus piezotolerans), WP_123017970.1 (Lacticaseibacillus paracasei), WP_152617979.1 (Caldibacillus thermoamylovorans), WP_206919644.1 (Enterococcus hulanensis), and WP_041556724.1 (Carnobacterium sp. 17-4).

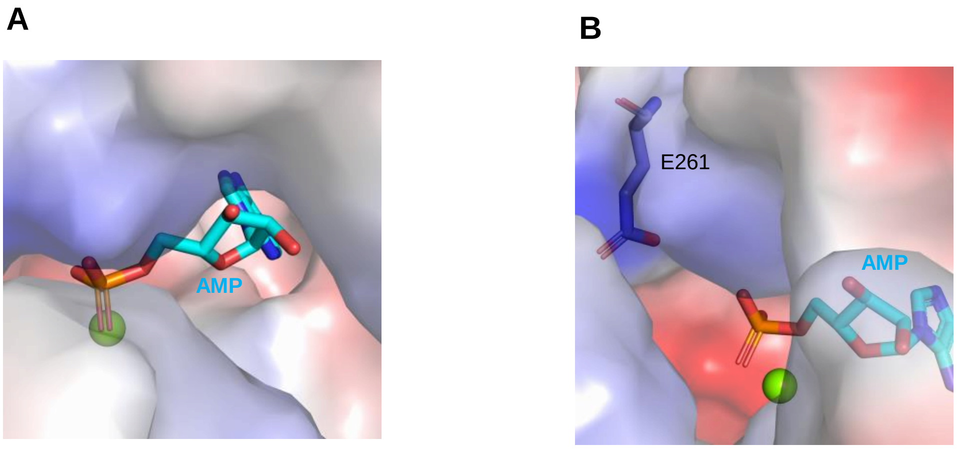

Figure S7. Electrostatic surface representation of FmnB-AMP. Positively charged residues are shown in blue, and negatively charged residues are shown in red. **A,** The AMP (in cyan) is buried in the hydrophobic pocket. **B,** The binding pocket of the phosphate group in FmnB-AMP is polarized by positive (blue) and negative (red) charges. The electrostatic potential was computed using the APBS tools in PyMol (http://www.pymol.org/).

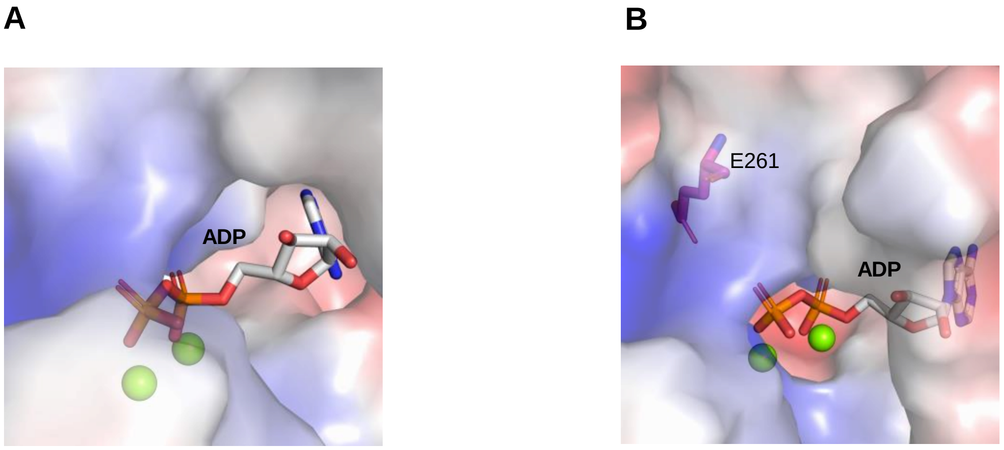

Figure S8. Electrostatic surface representation of FmnB-ADP. Positively charged residues are shown in blue, and negatively charged residues are shown in red. **A,** The ADP (in gray) is buried in the hydrophobic pocket. **B,** The binding pocket of the phosphate groups in FmnB-ADP is polarized by positive (blue) and negative (red) charges. The electrostatic potential was computed using the APBS tools in PyMol (http://www.pymol.org/).

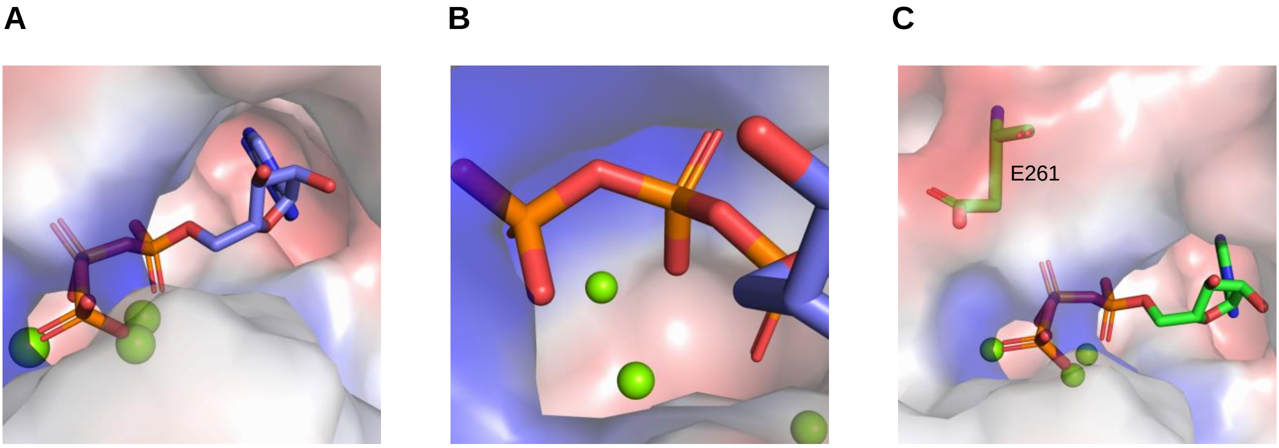

Figure S9. Electrostatic surface representation of FmnB-ATP. Positively charged residues are shown in blue, and negatively charged residues are shown in red. **A,** The ATP (in slate) is buried in the hydrophobic pocket. **B,** The binding pocket of the phosphate groups in FmnB-ADP is polarized by positive (blue) and negative (red) charges. **C,** The charge distribution for E261. The electrostatic potential was computed using the APBS tools in PyMol (http://www.pymol.org/).

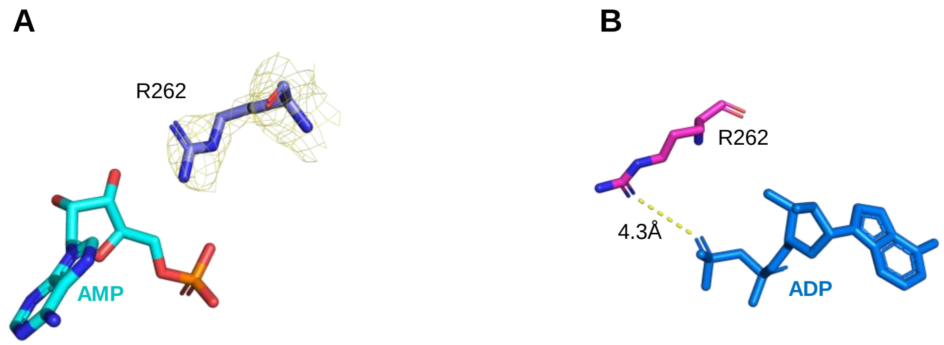

Figure S10. Structure of R262 in AMP- and ADP- bound FmnB. A, Electron density maps of R262 in FmnB-AMP. The 2Fo-Fc omit map of R262 contoured at 1.0σ is shown in yellow. **B,** Distance between R262 and ADP.

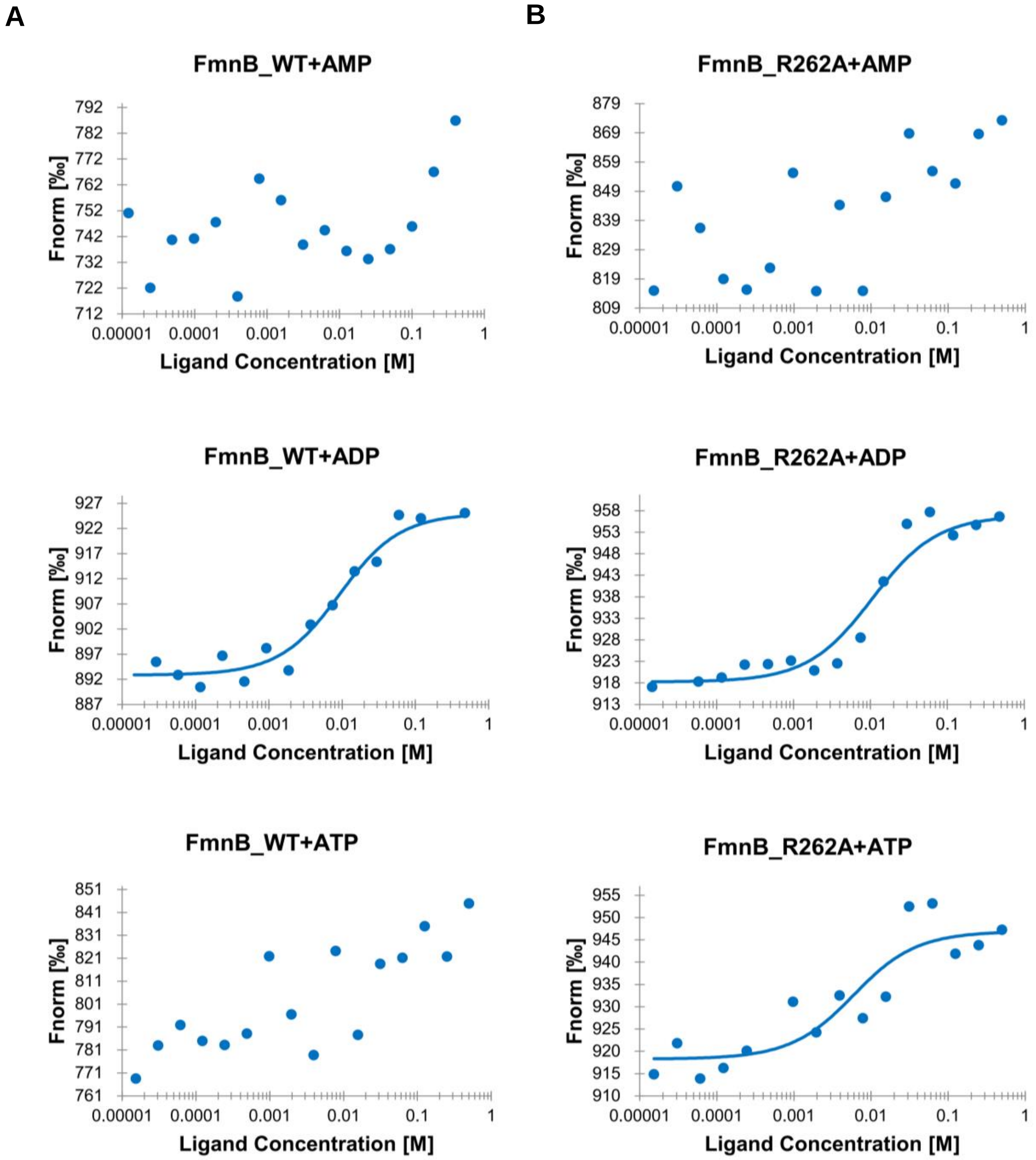

Figure S11. Measurement of FAD binding to wild-type FmnB and the R262 variant by MST. A, The Kd value between FmnB_WT and ADP was 8.32±1.69 mM. **B,** The Kd values between FmnB_R262A and ADP and ATP were 9.90±2.51 mM and 5.24±2.74 mM, respectively.

A

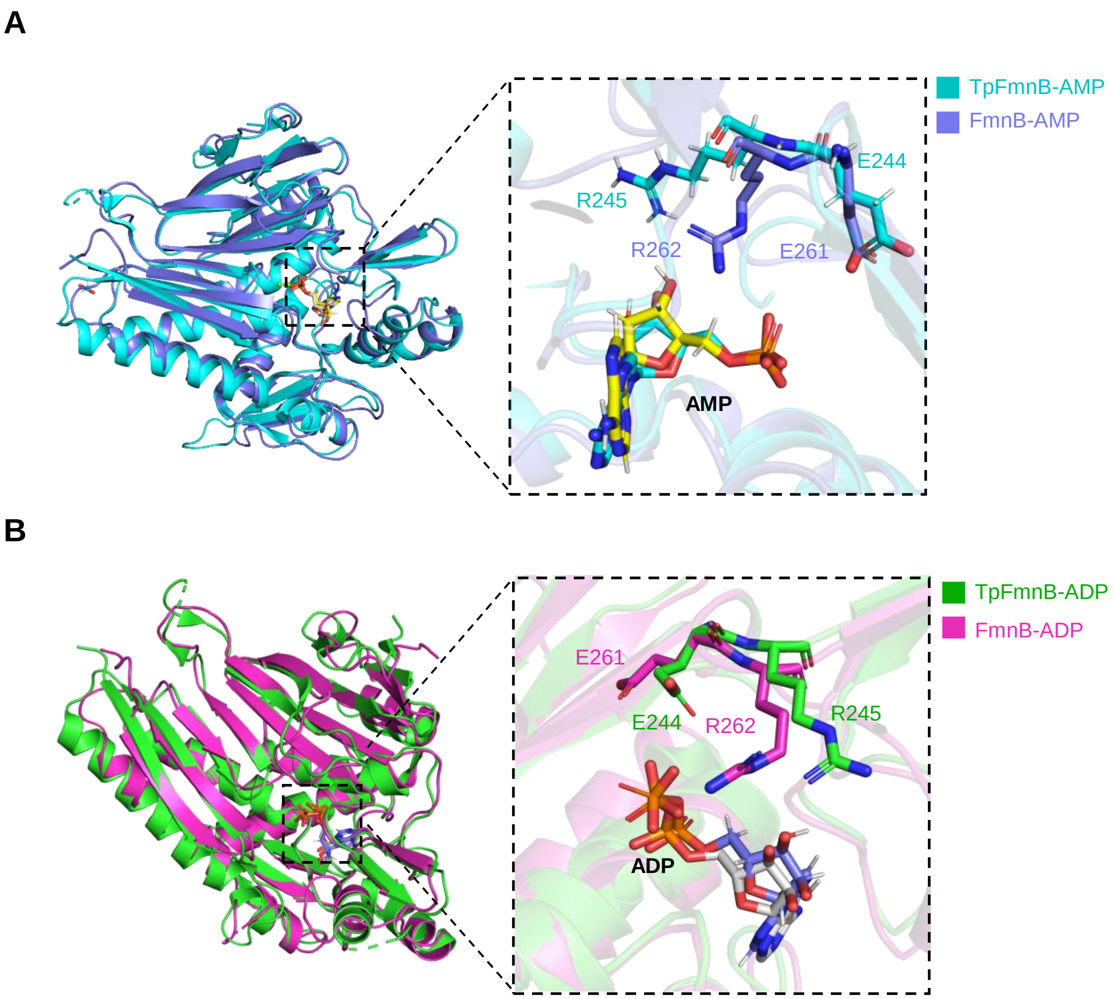

Figure S12. Comparison of AMP- and ADP- bound FmnB and TpFmnB. A, Superposition of FmnB-AMP (slate) and TpFmnB-AMP (cyan). **B,** Superposition of FmnB-ADP (magenta) and TpFmnB-ADP (green).

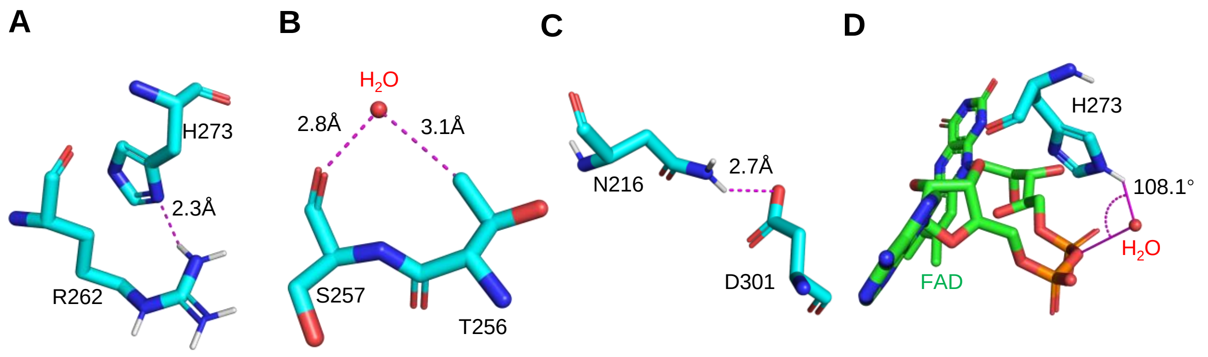

Figure S13. Stabilization of key residues in catalytic centers. A, Interaction between H273 and R262 in FmnB-FAD. The hydrogen bond is shown with magenta dashes. **B,** Interaction between T256 and S257 in FmnB-FAD. **C,** Interaction between N216 and D301 in FmnB-FAD. **D,** Angle of a water molecule between the phosphate group of FAD and H273 of FmnB. The angle is shown with magenta dashes.

Table S1. Data collection and refinement statistics.

	Apo-FmnB PDB ID: 7F39	FmnB-FAD PDB ID: 7ESA	FmnB-AMP PDB ID: 7ESC	FmnB-ADP PDB ID: 7F2U	FmnB-ATP PDB ID: 7ESB
**Data collection**					
Resolution range	36.45 - 1.89	36.28 - 1.8	39.46 - 2.2	27.58 - 1.984	31.37 - 1.703
Space group	P 2_1 2_1 2_1	P 2_1 2_1 2_1	P 2_1 2_1 2_1	P 1 2_1 1	P 2_1 2_1 2_1
Unit cell	65.913 66.768 87.493 90 90 90	66.0887 67.2939 86.7936 90 90 90	47.8749 67.872 97.0032 90 90 90	48.02 66.489 109.1 90 95.606 90	66.0887 67.2939 86.7936 90 90 90
CC1/2	1	0.971	0.995	0.993	0.98
Completeness (%)	99.69	99.97	99.21	91.55	98.83
Mean I/sigma(I)	418.20	48.17	16.63	11.68	31.76
Multiplicity	1.1	12.9	2.0	1.5	12.9
R-merge	0	0.1193	0.04471	0.04182	0.2341
**Refinement**					
Unique reflections	31500	36579	16541	43550	42584
R_{work}/ R_{free}	19.27/21.92	0.1942/22.27	24.47/28.20	20.38/24.82	17.08/19.67
Ramachandran favored (%)	98.75	98.12	96.56	97.17	98.72
Ramachandran allowed (%)	1.25	1.56	4.38	2.83	1.28
Ramachandran outliers (%)	0.00	0.00	0.00	0.00	0.00
